# Further validation of the efficacy of mesenchymal stem cell infusions for reducing mortality in COVID-19 patients with ARDS

**DOI:** 10.1038/s41536-021-00161-z

**Published:** 2021-09-09

**Authors:** Ashok K. Shetty, Padmashri A. Shetty, Gabriele Zanirati, Kunlin Jin

**Affiliations:** 1 Institute for Regenerative Medicine, Department of Molecular and Cellular Medicine, Texas A&M University College of Medicine, College Station, TX USA; 2 Ramaiah Medical College, Bengaluru, Karnataka India; 3 Brain Institute of Rio Grande do Sul (BraIns), Pontifical Catholic University of Rio Grande do Sul (PUCRS), Porto Alegre, Brazil; 4 Department of Pharmacology and Neuroscience, University of North Texas Health Science Center Fort Worth, Fort Worth, TX USA

**Keywords:** Respiratory distress syndrome, Mesenchymal stem cells

## Abstract

Recent double-blind, randomized, controlled trials reported that human umbilical cord-derived mesenchymal stem cell (MSC) infusions in COVID-19 patients with acute respiratory distress syndrome (ARDS) could diminish cytokine storm and lung damage. While these outcomes are significant, additional phase II/III trials are required to validate the efficacy of MSCs to improve the survival of COVID-19 patients with ARDS. Future studies also need to assess the efficacy of MSCs to prevent long COVID.

Coronavirus disease 2019 (COVID-19), a grave acute respiratory illness, is caused by severe acute respiratory syndrome coronavirus 2 (SARS-CoV-2)^1,2^. SARS-CoV-2 infection is a worldwide pandemic currently with >219 million infected cases and >4.5 million deaths. Over 40 million people have already been infected in the United States, and >659,000 people have died of COVID-19 [Worldometers.info]. SARS-CoV-2 infection initially causes mild-to-moderate symptoms, which include fever, cough, loss of smell and taste, muscle aches, fatigue, and shortness of breath, but severe cases with acute respiratory distress syndrome (ARDS), cytokine storm, or pneumonia result in considerable mortality^1,3,4^. Older individuals and individuals with certain health conditions have a higher risk of developing severe COVID-19 and mortality^5^. Overactive immune response causing cytokine storm and immunothrombosis are believed to underlie severe COVID-19^6,7^.

A widespread expression of the angiotensin-converting enzyme 2 (ACE2) receptor in the lung alveolar cells and capillary endothelial cells facilitates severe respiratory illness following SARS-CoV-2 infection^8,9^. Such infection results in a cytokine storm with elevations in multiple proinflammatory cytokines, leading to edema, air exchange dysfunction, ARDS, secondary infection, and death in many cases^8^. Because ACE2 expression is also present in other organs, including the heart, liver, kidney, and digestive system, infected patients develop other complications such as myocardial injury, arrhythmia, acute kidney injury, shock, and death from multiple organ dysfunction^8,10^. COVID-19 patients developing ARDS currently receive high-flow oxygen therapy, intensive care, and, frequently, mechanical ventilation^6,11–14^. Treating COVID-19 patients afflicted with ARDS, cytokine storm, or pneumonia is challenging despite several recent drug combinations showing variable efficacy^15–17^. On a positive note, vaccines against SARS-CoV-2 are now available, but it will take a significant amount of time to vaccinate the vast majority of the world population. While the vast majority of effort is currently aimed at reducing or eliminating infection through vaccination, there is also a need for reducing the mortality in severely ill patients with COVID-19. Therefore, safe and effective treatment for COVID-19 patients with severe complications such as ARDS, cytokine storm, or pneumonia is critical for saving lives.

Previously, studies from China and Mexico^8,18,19^ have shown that infusions of human umbilical cord-derived mesenchymal stem cells (UC-MSCs) into COVID-19 patients resulted in better functional outcomes^8,18–22^. Leng and associates showed that seven patients with COVID-19 pneumonia displayed improved functional outcomes and recovered after an intravenous administration of clinical-grade human UC-MSCs^8^. Among the patients who received UC-MSC infusions, one had a critically severe type, four had severe types, and the other two had common types of pneumonia^8^. This study provided the first evidence of the promise of MSC therapy for saving the lives of COVID-19 patients developing severe complications and has resulted in the commencement of many clinical trials^23^. A few subsequent studies also found that the infusion of UC-MSCs was safe in COVID-19 patients^19,22^. The study by Iglesias and colleagues enrolled five patients with severe ARDS who have not shown improvements in their clinical conditions with the prevailing medical care for 48 h and displayed arterial oxygen partial pressure (PaO_2_)/fractional inspired oxygen (FiO_2_) values <100 mmHg^19^. The study also reported that MSCs mediated anti-inflammatory effects in the lungs, evident from an improved respiratory function with higher PaO_2_/FiO_2_ values^19^. Treatment of MSCs resulted in the survival of three patients who were extubated 9 days post-infusion. However, the lack of double-blind, randomized, controlled trials using MSCs has raised questions about the efficacy of MSCs among skeptics.

While the outcome of many more extensive double-blind clinical trials for severe COVID-19 patients is yet to be published, Lanzoni and colleagues’ study has been recently published by the journal *Stem Cell Translational Medicine*^14^. This study represents the first double-blind, phase I/IIa, randomized, controlled trial of MSC treatment for COVID-19 patients. Table 1 lists the significant outcomes of double-blind, randomized controlled trials performed so far using MSCs. The double-blind study by Lanzoni and associates showed that infusions of UC-MSCs were safe for COVID-19 patients with complications, such as ARDS and cytokine storm^14^. The study also did not find any serious adverse events in the 12 patients receiving UC-MSC infusions. The patients in the control and MSC treatment groups were matched for age and ARDS severity, which balanced the control and MSC treatment groups. The study also revealed that the cytokine storm could be restrained through MSC infusions in COVID-19 patients with ARDS. MSC infusions did not decrease the viral load but significantly dampened the concentration of multiple proinflammatory cytokines. This study’s critical takeaway message is that the survival of COVID-19 patients with ARDS could be improved substantially with MSC treatment. In all, 91% survival was seen in the MSC infusion group compared to 42% survival in the control group. This is the most impressive finding from this controlled trial. Since severe COVID-19 is widely believed to be due to the overactive immune response with cytokine storm causing other complications such as immunothrombosis, mini-strokes, and multiple organ failure, the ability of MSCs to modulate the immune response and improve the survival of patients with ARDS is a significant advance. Overall, this trial’s findings validate the results of previous trials^8,19^. The results of another randomized, double-blind, placebo-controlled phase II trial using UC-MSCs in COVID-19 patients with lung damage have been published recently^24^. UC-MSC infusions (~40 million/infusion, 3 infusions over 6 days) in 49 patients resulted in a reduced lung lesion volume compared to 25 patients receiving the placebo^24^. These findings suggest that UC-MSC infusions could be employed as an adjunct therapy to the standard care of COVID-19 patients with lung damage.

**Table 1:** Double-blind, randomized, controlled trials using MSCs in COVID-19 patients.

Type of MSCs administered	Study design and reference	No. of patients in whom MSC infusion efficacy has been measured	Major inclusion criteria	Measured parameters	Major outcomes
Umbilical cord MSCs	Phase I/IIa (single center, double blind, randomized, and controlled)^14^	12	COVID-19 with ARDS and cytokine storm	Survival Inflammation	Improved survival Reduced proinflammatory cytokines
Umbilical cord MSCs	Phase II (multicenter, double blind, randomized, and controlled)^24^	49	COVID-19 with confirmed lung damage	Lung lesion volume 6-Min walk test	Reduced lung lesion volume Increased distance traveled

MSCs have been employed widely in cell therapy, which comprises numerous preclinical studies and a significant number of clinical trials^25–27^. The rationale for employing MSC infusions for COVID-19 complications is the safety and efficacy of MSCs for immune system-mediated inflammatory diseases, such as graft-versus-host disease^28^, type 2 diabetes^29^, and spinal cord injury^30^. Immunomodulatory effects of MSCs are believed to underlie improved function after MSC infusions in multiple disease conditions, as MSCs secrete multiple paracrine factors, which positively interact with immune cells, facilitating immunomodulation^25–27,31,32^. The better outcome in COVID-19 patients after MSC infusions also appeared to comprise the robust anti-inflammatory activity of MSCs suppressing the cytokine storm. MSCs typically accumulate in the lungs after intravenous infusion, which likely facilitates the secretion of multiple paracrine factors^33^, leading to significant protection and rejuvenation of alveolar epithelial cells, and improved lung function in COVID-19 patients. Figure 1 depicts the potential mechanisms by which MSCs improve outcomes in COVID-19 patients.

**Fig. 1:**
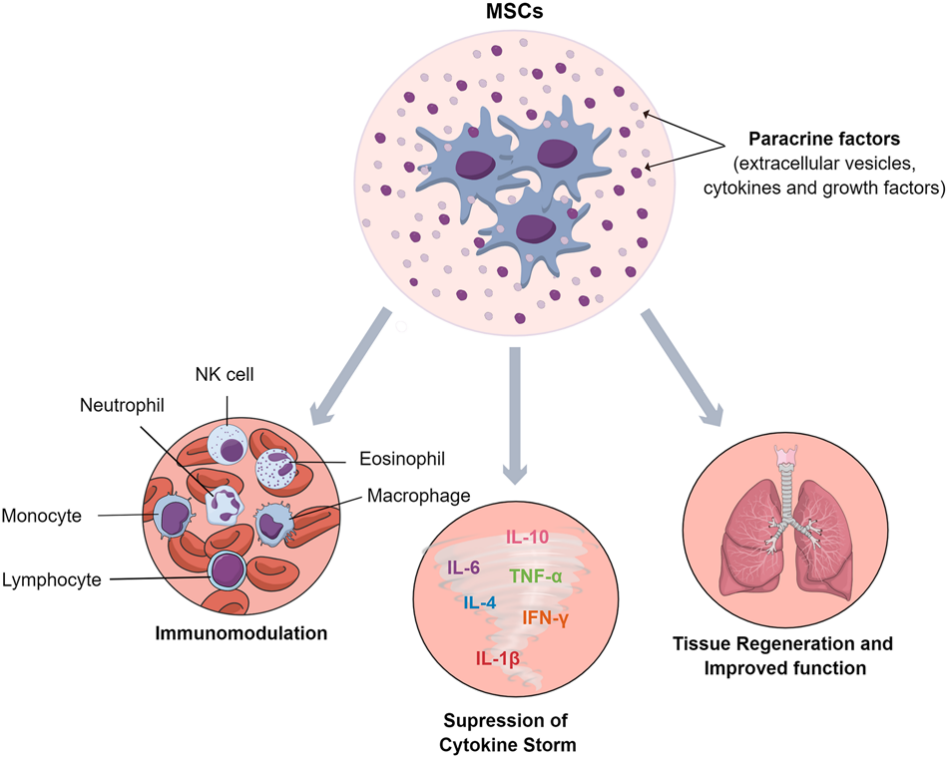
Potential mechanisms by which mesenchymal stem cells (MSCs) can improve outcomes in COVID-19 patients. MSCs can directly modulate a variety of immune cells from their proinflammatory states into non-inflammatory or anti-inflammatory phenotypes, suppress cytokine storm, and promote lung regeneration.

While the new clinical trial results provide further support for considering MSC infusions for COVID-19 complications, a few critical issues need to be addressed in future MSC therapy trials for COVID-19 patients with ARDS. The numbers of patients recruited in one of the trials were low (24 subjects; out of that, 12 patients received MSC infusions, and the other 12 were controls)^14^. However, another trial has validated some of the beneficial effects of MSC infusions in 49 COVID-19 patients^24^. Since UC-MSCs were used in both studies, as in the previous study^8^, it will be necessary to ascertain whether similar efficacy could be obtained from MSCs from other sources such as those derived from the bone marrow or adipose tissue. The average age of patients employed in these trials was ~59–60 years^14,24^. Therefore, it remains to be determined whether MSC infusions would be efficacious in much older COVID-19 patients (e.g., ≥65 years). Furthermore, the dose employed was 40–100 million per infusion, with infusions separated by 72 h apart. Dose–response studies are needed in the future to improve the efficacy further. Since the primary endpoint was safety, efficacy measures in these studies were restricted to patient survival and cytokine levels in the blood^14^ or lung lesion volume and a 6-min walk test^24^. Future studies need to assess whether MSC infusions in COVID-19 patients with ARDS or other complications would prevent the symptoms of long COVID. Patients with long COVID (i.e., patients with symptoms persisting for >6 months post-infection) experience persistent brain-related problems, including brain fog typified by cognitive impairments, post-exertional malaise, and chronic fatigue, mimicking chronic multisymptom conditions, such as fibromyalgia^34,35^, chronic fatigue syndrome^36^, or Gulf War Illness^37^. Additional phase II/III trials evaluating the outcomes of MSC infusions for reducing mortality and preventing long COVID symptoms are needed. Also, while the advent of efficacious vaccines has reduced SARS-CoV-2 infections in many nations, SARS-CoV-2-infected survivors experiencing long COVID symptoms might benefit from MSC therapy because of the ability of MSCs to ease neuroinflammation and promote regeneration in organs, such as the lungs, brain, and heart.

## Data Availability

All data needed to evaluate the conclusions of this commentary are present in the paper.
